# The Roles of the Anthraquinone Parietin in the Tolerance to Desiccation of the Lichen *Xanthoria parietina*: Physiology and Anatomy of the Pale and Bright-Orange Thalli

**DOI:** 10.3390/ijms25137067

**Published:** 2024-06-27

**Authors:** Amina G. Daminova, Ilya Y. Leksin, Venera R. Khabibrakhmanova, Oleg P. Gurjanov, Ekaterina I. Galeeva, Tatyana V. Trifonova, Ayrat R. Khamatgalimov, Richard P. Beckett, Farida V. Minibayeva

**Affiliations:** 1 Kazan Institute of Biochemistry and Biophysics, FRC Kazan Scientific Center, Russian Academy of Sciences, 420111 Kazan, Russia; daminova.ag@gmail.com (A.G.D.); lecsinilya@mail.ru (I.Y.L.); venerakhabirakhmanova@gmail.com (V.R.K.); gurjanov58@gmail.com (O.P.G.); kgu@mail.ru (E.I.G.); trifonovatatyana@yandex.ru (T.V.T.); 2 Arbuzov Institute of Organic and Physical Chemistry, FRC Kazan Scientific Center, Russian Academy of Sciences, 420088 Kazan, Russia; ayrat_kh@iopc.ru; 3 School of Life Sciences, University of KwaZulu-Natal, PBag X01, Scottsville 3209, South Africa; rpbeckett@gmail.com; 4 “Biomarker” Research Laboratory, Institute of Fundamental Medicine and Biology, Kazan Federal University, 420008 Kazan, Russia

**Keywords:** parietin, lichen, desiccation, thallus anatomy, photosynthesis, lipid peroxidation, membrane stability index, extractable substances, thermal decomposition

## Abstract

Lichens are symbiotic organisms that effectively survive in harsh environments, including arid regions. Maintaining viability with an almost complete loss of water and the rapid restoration of metabolism during rehydration distinguishes lichens from most eukaryotic organisms. The lichen *Xanthoria parietina* is known to have high stress tolerance, possessing diverse defense mechanisms, including the presence of the bright-orange pigment parietin. While several studies have demonstrated the photoprotective and antioxidant properties of this anthraquinone, the role of parietin in the tolerance of lichens to desiccation is not clear yet. Thalli, which are exposed to solar radiation and become bright orange, may require enhanced desiccation tolerance. Here, we showed differences in the anatomy of naturally pale and bright-orange thalli of *X. parietina* and visualized parietin crystals on the surface of the upper cortex. Parietin was extracted from bright-orange thalli by acetone rinsing and quantified using HPLC. Although acetone rinsing did not affect PSII activity, thalli without parietin had higher levels of lipid peroxidation and a lower membrane stability index in response to desiccation. Furthermore, highly pigmented thalli possess thicker cell walls and, according to thermogravimetric analysis, higher water-holding capacities than pale thalli. Thus, parietin may play a role in desiccation tolerance by stabilizing mycobiont membranes, providing an antioxidative defense, and changing the morphology of the upper cortex of *X. parietina*.

## 1. Introduction

Lichens are one of the most successful symbiotic associations, comprising an ascomycete fungus, the “mycobiont”, associated with a “photobiont”, mostly green algae (“chlorobionts”), and/or cyanobacteria (“cyanobionts”) [1,2,3]. Lichens can survive in harsh environments characterized by high levels of stress such as dehydration, temperature extremes, and solar radiation [4,5,6,7]. The synthesis of secondary metabolites is often considered to play an important role in the tolerance of lichens to stress [8]. Many studies on secondary metabolites produced by mycobionts have centered on their potential medicinal applications, and they have been shown to display a variety of antibiotic, antimycotic, or antiviral properties [9,10,11]. For example, the bright-orange anthraquinone parietin (also termed physcion) produced is the predominant cortical pigment of lichens in the genera *Caloplaca* and *Xanthoria* but can also been found in Angiosperms such as in the roots of *Rumex crispus* [12]. Parietin is characterized by the low solubility in water, aggregation potential, and photoinstability [13]. Parietin in *Xanthoria parietina* (Ascomycota, Teloschistaceae) has been reported to have antioxidative, antibacterial, and anti-tumor properties [13,14,15]. *Xanthoria parietina* is a globally distributed epiphytic species that prefers nitrogen-rich habitats such as agricultural land but also occurs in urban and industrial areas. The lichen grows relatively fast [16,17], developing a foliose thallus that varies in color from pale green to bright orange. Thallus pigmentation increases with exposure to solar radiation, being conspicuously more pigmented in sunny microhabitats [18,19]. The characteristic orange color of the lichen thallus is due to the presence of crystals of parietin that occur mostly in the upper cortex [20]. In general, *Xanthoria parietina* appears to be more resistant to environmental pollution than most other lichens [21,22,23,24], contributing to the interest in this lichen and parietin, its major secondary metabolite. The roles of parietin as a protective metabolite against high photosynthetic active radiation (PAR) and ultraviolet B (UV-B) are widely recognized [25]. Furthermore, good evidence has been presented that parietin protects *X. parietina* from Cd toxicity by reducing Cd-induced oxidative stress [26] and has also been reported to promote the recovery of the thalli after the stress exposure to very high ozone concentrations [27]. However, very little is known about the potential contribution of parietin to the tolerance of *X. parietina* to desiccation tolerance. Intuitively, it could be predicted that thalli that have become bright orange, having been exposed to at least moderate solar radiation, may need additional desiccation tolerance. We hypothesized that although parietin, as an anthraquinone, is a hydrophobic compound, it can contribute to the hygroscopicity of the upper cortex, which determines the amount of extracellular medullary water in the interior of the thallus [28]. Therefore, the aim of the present work was to explore the possible contribution of parietin in desiccation tolerance of the lichen *X. parietina*. For this purpose, membrane stability, lipid peroxidation, photosystem II (PSII) activity, relative water content (RWC), thallus anatomy, and topography were compared between pale-green, bright-orange, and bright-orange thalli of *X. parietina*, with parietin removed using the “acetone-rinsing” technique during the desiccation/rehydration cycle. In addition, the yield of extractable substances (ES), parietin content, and thermal decomposition were also measured in different types of thalli.

## 2. Results and Discussion

Lichens are extremophiles, which colonize stressful environments and survive in the most extreme and inhospitable habitats [29]. Among the many mechanisms that allow them to thrive even in such conditions, it is believed that secondary metabolites, including pigments, can protect lichen thalli from stress [30]. The photoprotective properties of the orange-yellowish-colored pigment parietin of an anthraquinone nature have been widely studied. Parietin synthesized by the mycobiont protects a photobiont against strong UV radiation [20,31] and high PAR [30]. Parietin is particularly effective at absorbing blue light. Its concentration in lichen thalli varies seasonally, and this generally correlates with solar radiation [32]. It seems likely that bright-orange thalli will have been exposed to considerably more solar radiation than pale thalli and may therefore require enhanced desiccation tolerance. However, the role of parietin in the protection of lichens against desiccation has not been investigated.

**Figure 1:**
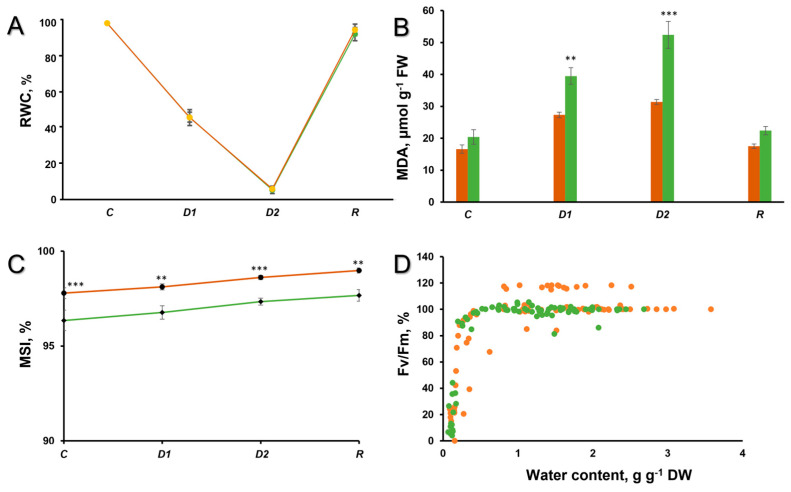
(**A**) Relative water content, (**B**) Lipid peroxidation, and (**C**) Membrane stability index in the hydrated control (*C*), after 50% desiccation (*D1*), after 90% desiccation (*D2*), and rehydration (*R*) of the thalli *X. parietina*. (**D**) F_V_/F_M_ during desiccation. Absolute values of F_V_/F_M_ in the control were 0.739 ± 0.050 for bright-orange thalli and 0.724 ± 0.017 for acetone-rinsed thalli. For all graphs, orange designates bright-orange thalli, while green designates acetone-rinsed thalli. Values are plotted as means ± SE, statistically significant differences are indicated as *p* ≤ 0.01 (**), ≤ 0.001 (***).

In the present study, the role of parietin in the response of the lichen *X. parietina* to desiccation and rehydration was tested by harmlessly removing parietin from pre-desiccated thalli using the “acetone-rinsing” technique. This technique allows comparison of the effects of stress on thalli with and without parietin, as reported for other lichen substances by Ndhlovu et al. (2024) [33]. Hydrated bright-orange and acetone-rinsed lichen thalli were kept in a desiccator over a saturated CaCl_2_ solution for 4 and 14 h. Rates of water loss from both types of thalli were similar, and after desiccation for 4 h, the RWC dropped to ca. 50% RWC (D1), and after 14 h to ca. 10% RWC (D2) (Figure 1A). Subsequent rehydration for 1 h resulted in almost full recovery of RWC (Figure 1A). Interestingly, during slower desiccation over a saturated NaCl solution, water loss by bright-orange thalli was slightly slower than from acetone-rinsed thalli during the first 6 h, although further water losses (until 24 h) occurred at similar rates in both types of thalli (Figure S1). Assessment of the photosynthetic efficiency demonstrated that removal of parietin from pre-desiccated thalli had very little effect on photosynthetic activity, and the relative values of F_V_/F_M_ in bright-orange and acetone-rinsed thalli declined in a similar way during desiccation. However, the absolute values of the PSII activity were slightly lower in the acetone-rinsed lichen thalli (Figure 1D). In contrast, Ndhlovu et al. (2024) found that in other lichen species (containing secondary metabolites other than parietin), substance removal generally reduced the effects of desiccation on PSII activity [33], probably due to the very high antioxidant activity of lichen substances [34,35]. However, in *X. parietina*, there were differences in the overall thallus level of lipid peroxidation, a stress parameter predominantly indicative of the mycobiont. Lipid peroxidation (assessed as malondialdehyde (MDA) content) in acetone-rinsed thalli increased significantly during desiccation, while in the bright-orange thalli, MDA increased only slightly during desiccation (Figure 1B). After rehydration, the level of lipid peroxidation dropped to initial values in both types of thalli (Figure 1B). We suggest that the antioxidative properties of parietin [36] may have reduced the level of lipid peroxidation, thereby improving the membrane stability of *X. parietina* during desiccation.

The membrane stability index during the desiccation/rehydration cycle remained higher in bright-orange thalli than in non-pigmented thalli (Figure 1C). These results are similar to those of Ndhlovu et al. (2024), where secondary metabolite removal generally increased membrane leakage during rehydration following desiccation [33].

**Figure 2:**
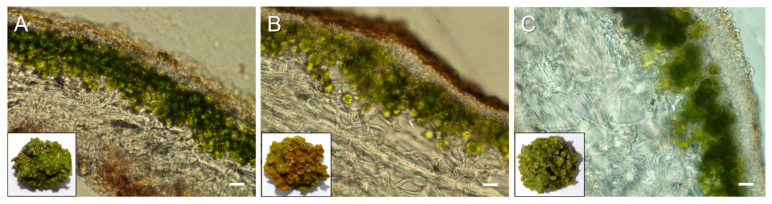
The cross sections of pale (**A**), bright-orange (**B**), and acetone-rinsed (**C**) thalli of *X. parietina*. Bar = 25 μm. Inserts represent the photographs of the whole thalli.

The morphology of the upper cortex in the cross sections of pale, bright-orange, and acetone-rinsed thalli of *X. parietina* was viewed with light microscopy (Figure 2). In pale thalli of *X. parietina*, weakly pigmented cells were visualized in the uppermost paraplectenchymatous cortex; underneath, there were non-pigmented leptodermous fungal cells followed by the algal layer comprising *Trebouxia* sp. (Figure 2A). In bright-orange thalli, the surface of the upper cortex appeared dark-brown-colored (Figure 2B), whereas in acetone-rinsed thalli, it was transparent and only slightly colored (Figure 2C). The position of the parietin is consistent with it being produced by the mycobiont and acting as a protecting agent against high and harmful PAR and UV-B [37].

**Figure 3:**
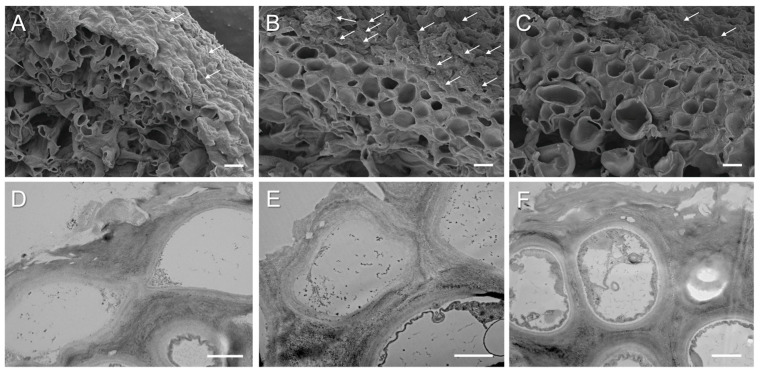
SEM and TEM images of cross sections from pale (**A**,**D**), bright-orange (**B**,**E**), and acetone-rinsed (**C**,**F**) thalli of *X. parietina*. White arrows indicate needle-shaped crystals of parietin. Bar (**A**–**C**) = 2 µm, (**D**–**F**) = 1 µm.

SEM and TEM were used to visualize the microstructure of cross sections of pale, bright-orange, and acetone-rinsed thalli of *X. parietina*. The relief of the anticlinal cell walls of the upper cortex of lichen thalli was heterogeneous, with depressions and bulges containing needle-shaped crystals (Figure 3A,B). The majority of lichen secondary metabolites typically occur as hydrophobic crystals on the hyphal cell walls and are generally extremely insoluble in water [38]. In *X. parietina*, the upper cortex of the bright-orange thalli (Figure 3B) was thicker than that of the pale thalli (Figure 3A). In addition, in the upper cortex, the diameter of pores representing the lumens of fungal hyphae per unit of the cross-sectional area in the pale thalli (Figure 3A) was ca. 1.6 times greater than that in the bright-orange thalli (Figure 3B). After acetone rinsing of the bright-orange thalli, the thickness of the cell walls did not significantly change (Figure 3C). Analysis of the ultrastructure of mycobiont cells showed that secondary metabolites accumulated in the pale, bright-orange, and acetone-rinsed thalli; however, in the bright-orange thalli, the amount of inclusions, probably containing parietin, inside the hyphae and between the lumens was higher. In the acetone-rinsed thalli, inclusions were observed in the inner layer between lumens of hyphae. The accumulation of parietin inside cells and between lumens of the hyphae in *X. parietina* probably occurs due to accumulation of the sugar ribitol that is synthesized by the photobiont and passed to the mycobiont [39]. Ribitol is a precursor for parietin, which is stored in the cortex-forming hyphae in two forms in equal amounts: the intracellular parietin and the extracellular, crystalline parietin [39]. Interestingly, in the study by Jung et al. (2024), infection of terricolous lichen *Caloplaca* s.l. in the Coastal Range of the Atacama Desert with lichenicolous fungus led to a conspicuous blackening of this orange thalli [40]. This happens as a result of a loss of the lichen’s photosynthetic activity and an inhibition of the parietin synthesis as a shared pathway between the photobiont and the mycobiont, including a shift of secondary metabolism products. It can be suggested that parietin loss may have increased the susceptibility of the lichens to photoinhibition and desiccation stress.

**Figure 4:**
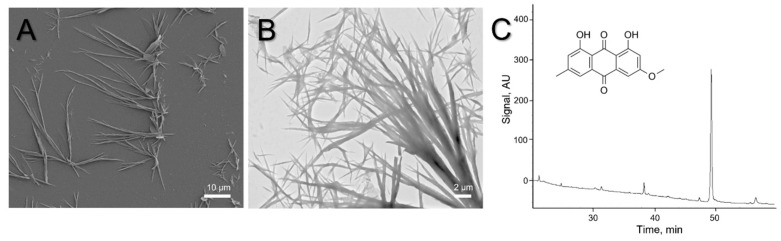
Visualization of parietin crystals using SEM (**A**) and TEM (**B**). HPLC elution profile of acetone extract from *X. parietina* with a major peak corresponding to parietin (**C**). Inclusion: the chemical structure of parietin (1,8-dihydroxy-3-methoxy-6-methyl-9,10-anthraquinone).

Microscopy observations of dried acetone extract demonstrated the presence of irregular needle-shaped crystals (Figure 4A,B). The morphology of parietin extracted from *X. parietina* was similar to the morphology of commercial parietin in the bright-yellow acetone solution that also appeared as needle-shaped crystals [41]. The identity of parietin was confirmed by high-performance liquid chromatography (HPLC) analysis; the major peak elution profile of the acetone extract corresponded to that of the parietin standard (Figure 4C).

Quantitation of parietin in the acetone extracts from the pale and bright-orange thalli showed an expectedly higher level of this pigment in the orange thalli compared to the pale thalli, while the yield of the total ES was slightly higher from the pale thalli (Table 1). We detected that desiccation of the thalli caused an opposite effect on the ES yield and the content of parietin in the pale and bright-orange thalli. While the ES yield from desiccated pale thalli increased, the ES yield from desiccated bright-orange thalli significantly (up to 50%) decreased. It can be suggested that not only extracellular but also intracellular metabolites are extracted from desiccated pale thalli. This can result from the membrane damage caused by oxidative stress during desiccation (Figure 1B). The presence of parietin in the bright-orange thalli prevents the leakage of substances from dry thalli; however, more experimental evidence is necessary to confirm this suggestion. The content of parietin decreased in desiccated pale thalli and slightly increased in desiccated bright-orange thalli (Table 1). After subsequent rehydration, regardless of the degree of thalli pigmentation, a significant decrease in the content of ES was observed. The level of parietin recovered in the orange thalli and remained quite low in the pale thalli.

Possibly, the pale thalli were generally less stress-tolerant and, compared to pigmented thalli, had lower activities or levels of antioxidative enzymes. When desiccation stressed in pale thalli, parietin may have acted as an important antioxidant, explaining why levels fell so much. In contrast, bright-orange thalli, being exposed to solar radiation for a long time, are more stress-tolerant due to accumulation of parietin and osmoprotective metabolites such as polyols and glycerol, higher antioxidative defense and morphological changes in the thallus structure.

The analysis of thermogravimetry (TG) and derivative of TG data (DTG) curves showed that thermal decomposition characteristics of all lichen samples had similar character with two stages of mass loss (Figure S2). The first stage, estimated as ~4.8–6.6% of mass loss, was observed within the range of 82–85 °C (Table 2) and accompanied by corresponding endothermal peaks at 86.0–86.9 °C (Figure S2). Most likely, this stage can be explained by the water loss, and therefore, following heating up to 100 °C, the bright-orange thalli of *X. parietina* lose more water than pale thalli. The second stage, estimated as ~61.6–69.8% of mass loss, was observed within the range of 313–316 °C (Table 2) and accompanied by corresponding endothermal shoulders at ~315–320 °C (Figure S2).

This stage is characterized by degradation of the structural components of cell walls such as hemicellulose and cellulose [42]. Interestingly, further heating over 400 °C resulted in a slower degradation of the components of acetone-rinsed thalli than those of the thalli with parietin (Figure S2). These data suggest that the presence of parietin supports an improved water milieu and structural organization in the bright-orange thalli of *X. parietina*.

## 3. Materials and Methods

### 3.1. Sample Preparation

Pale and bright-orange thalli of the lichen *Xanthoria parietina* (L.) Beltr. were collected from shaded and sunny locations in the Aishinsky forestry in the outskirts of Kazan, Russia (55°53 21.3 N 48°38 14.3 E). The material was cleaned, slowly dried at room temperature at a relative humidity of 60–70%, and then stored at −20 °C. Parietin was removed using the “acetone rinsing” technique [43,44]. Briefly, bright-orange thalli were desiccated overnight above silica gel and then gently shaken in 50 mL of acetone for 5 min three times. Thalli were air-dried at room temperature overnight to allow acetone evaporation. Acetone extracts were merged and further used for analyses (see below).

Before the start of the experiments, pale, bright-orange, and acetone-rinsed thalli were hydrated by placing them on moist filter paper in a climate chamber at 15 °C for 24 h. Hydrated thalli were desiccated in the desiccator over a saturated solution of CaCl_2_ for 4 and 14 h. RWC was estimated during the desiccation time course for 4 h (D1), 14 h (D2), and rehydration in liquid water for 1 h (R) according to the protocol described in [45]. To measure photosynthetic parameters, slower desiccation over a saturated solution of NaCl was used.

### 3.2. Chlorophyll Fluorescence

The chlorophyll fluorescence was measured using a PAM (Pulse Amplitude Modulation) fluorometer (Hansatech FMS1, Hansatech Instruments, Kings Lynn, UK), as described by Ndhlovu et al. (2024) [33]. Briefly, after a dark adaptation period of at least 10 min, a flash of saturating light (8000 µmol m^−2^ s^−1^ for 0.8 s) was given, and F_V_/F_M_ was measured (F_V_ = variable fluorescence; F_M_ = maximum fluorescence). After the fluorescence level decreased to the initial dark value (Fo), the actinic light was switched on with a flux density of 25 µmol m^−2^ s^−1^, and after 1 min, a second saturating pulse was turned on to determine the maximum fluorescence yield (F’_M_) in the light-adapted state.

### 3.3. Lipid Peroxidation and Membrane Stability Index

Lipid peroxidation and the membrane stability index were measured in the hydrated control (C), after desiccation of thalli to a RWC of 50% (D1) and a RWC of 10% (D2) and during rehydration (R). The level of lipid peroxidation was assessed by accumulation of MDA [46]. Lichen thalli (0.5 g) were homogenized with 1.5 mL of 20% trichloroacetic acid solution. The optical density of supernatants was measured using a spectrophotometer UV-1600 (Shimadzu, Japan) at 532 nm, and the MDA content was estimated using the molar extinction coefficient of 156 mM^−1^ cm^−1^. Membrane stability was assessed by measuring electrolyte leakage from lichen thalli of *X. parietina* using a standard protocol [47]. Discs of lichen thalli were immersed in bidistilled Milli-Q water and kept at room temperature for 30 min. The electrical conductivity of the solution after incubation (C_1_) was measured using an Ohaus ST3100C-B conductometer (Pine Brook, NJ, USA). The total residual amount of electrolytes (C_2_) was determined by the electrical conductivity of the same solution after exposure of the same lichen disks to 100 °C for 30 min. The membrane stability index (MSI) was calculated using the equation: MSI = (1 − (C_1_ − C_0_)/(C_2_ − C_0_)) × 100%, where C_0_ is the electrical conductivity of bidistilled Milli-Q water.

### 3.4. Light, Scanning (SEM), and Transmission Electron Microscopy (TEM)

For microscopy, fragments of the thalli were embedded in 3% agarose blocks and sliced using a vibratome (Leica VT 1000S, Wetzlar, Germany), resulting in sections 50 µm thick. The anatomy and morphology of the lichen were visualized using light microscopy, SEM, and TEM. Firstly, the sections of lichen thalli were viewed using an epifluorescence microscope (Leica DM1000, Leica Biosystems, Wetzlar, Germany) with a digital camera at a magnification of 40×. Secondly, for SEM and TEM observations, the cross sections of the thalli of *X. parietina* were subsequently fixed in 2.5% glutaraldehyde in 0.1 M of Na-phosphate buffer, pH 7,4 and 1% OsO_4_, and dehydrated as described by [48]. Sections of lichen thalli or 10 µL of acetone extract were placed onto a glass sputter-coated with gold using the Q150T ES Coater (Quorum Technologies, Lewes, UK) and viewed using a high-resolution scanning electron microscope (Merlin, Carl Zeiss, Oberkochen, Germany) at a voltage of 5 kV. To assess the relative thickness of hyphal cell walls in lichen thalli, the number of hyphae per unit on the cross-sectional area of SEM images was determined using the ImageJ 1.48v program.

For TEM, samples were ultrathin-sectioned using an ultramicrotome (Leica, Wetzlar, Germany). Sections (50 nm thick) were stained with 2% aqueous uranyl acetate (*w*/*v*) for 20 min and Reynolds’ lead citrate for 7 min [49]. To image parietin, 10 µL of acetone extract was placed onto C-coated 200-mesh copper grids, and acetone was allowed to evaporate. Sections and parietin crystals were examined using an Excellence TEM (Hitachi HT 7700, Tokyo, Japan) at an accelerating voltage of 100 kV.

### 3.5. High-Performance Liquid Chromatography

The content of parietin was analyzed by HPLC using LicArt 62 chromatograph (Labconcept, Saint Petersburg, Russia). Lichen thalli were ground to pieces of 2–5 mm. Acetone (20 mL) was added to the samples (1 g) and incubated for 30 min at room temperature. Then, the supernatant was separated, and acetone (20 mL) was added to the precipitate; the extraction was repeated four times. The resulting supernatants were merged and dried using a rotary evaporator RV 10V (IKA, Staufen, Germany). The yield of extractive substances (ES) was assessed gravimetrically [50]. Separation was performed using a reverse-phase column Inertsil ODS-3 (3 µm, 4.6 × 250 mm (GL Sciences, Tokyo, Japan)). HPLC elution solutions were used as follows: eluent A—methanol–formic acid–water (30:1:69), eluent B—methanol with gradient: 0–1 min A—100%; 1–15 min A—40% and B—60%; 15–60 min B—100%. The flow rate of the eluent was 0.5 mL min^−1^. The identity of parietin was confirmed by comparison with the retention time and UV electron spectrum of commercial parietin (Cayman Chemical Company, Ann Arbor, MI, USA, purity ≥ 98%). The parietin content in the acetone extracts was determined using a calibration curve (30–500 µg ml^−1^ parietin, correlation coefficient (R) = 0.99).

### 3.6. Thermogravimetry/Differential Scanning Calorimetry

A combined method of Thermogravimetry/Differential Scanning Calorimetry (TG/DSC, NETZSCH STA 449 F3, Selb, Germany) was used for the synchronous thermal analysis of lichen thalli. An approximately 4.6–6.1 mg sample was placed in an Al crucible with a hole in the lid and heated from 25 °C to 600 °C. The same empty crucible was used as a reference sample. The analyses were carried out using a high-purity argon with a gas flow rate of 50 mL min^−1^. TG/DSC measurements were performed at the heating rates of 10 K min^−1^.

### 3.7. Statistical Analysis

The experiments were carried out in three biological and at least three analytical replicates. The results of the experiments were statistically processed and presented as arithmetic means with standard errors (SE). All experimental data have a normal distribution of the trait. One-way analysis of variance (ANOVA) was used with assessment of pairwise differences using Tukey and Bonferroni tests.

## 4. Conclusions

The present study provides insights into possible roles of parietin in the tolerance of lichens to desiccation. Here, we show that the accumulation of the orange pigment parietin in the upper cortex of the thallus influences the response of the lichen *X. parietina* to desiccation. Parietin removal does not have a significant effect on the rates of water loss and decline in PSII activity that occur during desiccation. However, the absence of parietin increases lipid peroxidation and reduces membrane stability during desiccation. Furthermore, highly pigmented thalli possess thicker cell walls and, according to thermogravimetric analysis, higher water-holding capacities than pale thalli. Thus, parietin may play a role in the desiccation tolerance of the mycobiont by stabilizing membranes, providing an antioxidative defense, and changing the morphology of the upper cortex of *X. parietina*. Future studies with extracted parietin crystals may help us to unravel the subtle relations between water molecules and this hydrophobic metabolite linked to the formation of extracellular water milieu in the interior of *X. parietina* thalli.

## Figures and Tables

**Table 1:** Content of extractable substances (ES) and parietin in acetone extracts of pale and bright-orange thalli of *X. parietina* in hydrated control (C), after 90% desiccation (D2) and rehydration (R).

Thallus	Treatment	ES Yield, mg g^−1^ DM	Parietin Content, mg g^−1^ DM
Pale	C	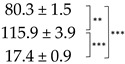	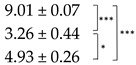
	D2		
	R		
Bright-Orange	C	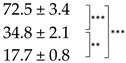	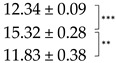
	D2 R		

Values are plotted as means ± SE, statistically significant differences are indicated as *p* ≤ 0.1 (*), ≤ 0.01 (**), ≤ 0.001 (***).

**Table 2:** Characteristics of thermal decomposition of the pale, bright-orange, and acetone-rinsed thalli of *Xanthoria parietina*.

Thallus	Stages of Mass Loss		Residual Mass, %
	I	II	
	TG, %/DTG, °C	TG, %/DTG, °C	
Pale	5.9/83.4	61.6/312.5	32.5
Bright-orange	6.6/82.0	69.8/315.7	23.6
Acetone-rinsed	4.8/85.2	65.0/313.9	30.2

## Data Availability

Data are contained within the article and Supplementary Materials.

## Supplementary Materials

The following supporting information can be downloaded at: https://www.mdpi.com/article/10.3390/ijms25137067/s1.
