# Supplementary material for: The Roles of the Anthraquinone Parietin in the Tolerance to Desiccation of the Lichen Xanthoria parietina: Physiology and Anatomy of the Pale and Bright-Orange Thalli
Source: Int J Mol Sci. 2024 Jun 27;25(13):7067. doi: 10.3390/ijms25137067 (PMC11240919; doi:10.3390/ijms25137067)
Supplement: Supplementary file 1 [file ijms-25-07067-s001.zip › ijms-3061918-supplementary.pdf]

Daminova et al.

The Roles of the Anthraquinone Parietin in the Tolerance to Desiccation of the Lichen *Xanthoria parietina*: Physiology and Anatomy of the Pale and Bright-Orange Thalli

Supplementary Materials:

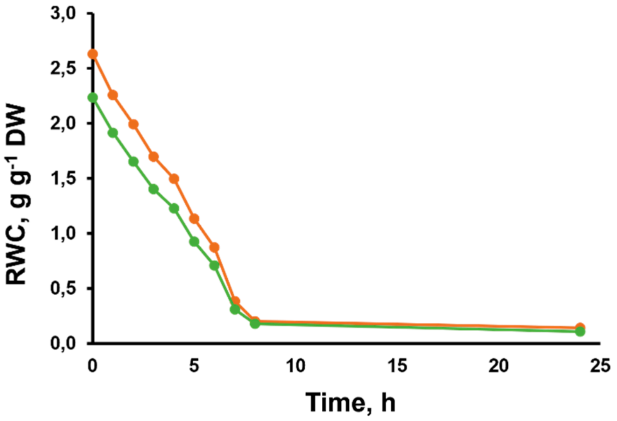

Figure S1. Relative water content of the thalli *X. parietina* during slow desiccation over a saturated NaCl solution. Orange colour designates bright orange thalli, while green colour designates acetone-rinsed thalli.

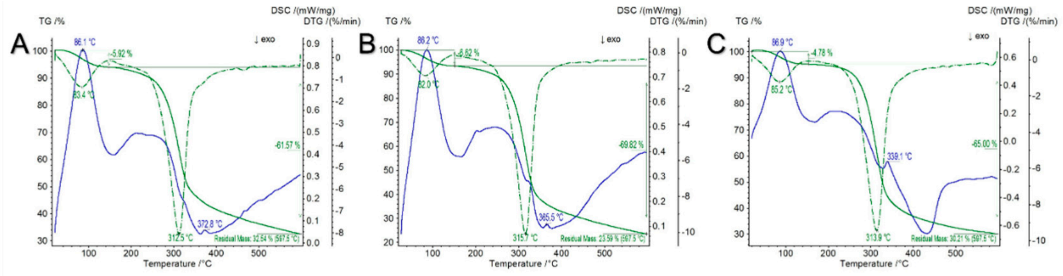

Figure S2. Thermal decomposition characteristics of pale (A), bright-orange (B) and acetone-rinsed (C) thalli of *X. parietina*: TG (green line), DTG (dotted green line), DSC (blue line).
